# Chinese herbal compound prescriptions combined with Chinese medicine powder based on traditional Chinese medicine syndrome differentiation for treatment of chronic atrophic gastritis with erosion: a multi-center, randomized, positive-controlled clinical trial

**DOI:** 10.1186/s13020-022-00692-7

**Published:** 2022-12-22

**Authors:** Tai Zhang, Beihua Zhang, Jinkang Xu, Shunping Ren, Shaogang Huang, Zhaohong Shi, Shaoju Guo, Liqun Bian, Ping Wang, Fengyun Wang, Yidong Cai, Xudong Tang

**Affiliations:** 1 Xiyuan Hospital, China Academy of Chinese Medical Sciences, Beijing, China; 2 Department of Gastroenterology, Xiyuan Hospital, China Academy of Chinese Medical Sciences, Beijing, China; 3 Institute of Digestive Diseases, Xiyuan Hospital, China Academy of Chinese Medical Sciences, Beijing, China; 4 Department of Gastroenterology, Kunshan Traditional Chinese Medicine Hospital, Kunshan, China; 5 Department of Gastroenterology, The Hospital of Shanxi University of Chinese Medicine, Taiyuan, China; 6 Department of Gastroenterology, The Second Affiliated Hospital of Guangzhou University of Chinese Medicine, Guangzhou, China; 7 Department of Gastroenterology, Wuhan No. 1 Hospital, Wuhan, China; 8 Department of Gastroenterology, Shenzhen Traditional Chinese Medicine Hospital, The Fourth Clinical Medical College of Guangzhou University of Chinese Medicine, Shenzhen, China

**Keywords:** Traditional Chinese medicine, Chronic gastritis with erosion, Multi-center, randomized study, Endoscopy

## Abstract

**Background:**

In this study, Chinese herbal compound prescriptions combined with Chinese medicine powder were evaluated for the treatment of chronic atrophic gastritis with erosion.

**Methods:**

This multi-center, randomized, positive drug control clinical trial randomly assigned 216 patients with chronic atrophic gastritis with erosion to three groups: (1) control group: aluminum plus magnesium suspension thrice per day for 4 weeks; (2) test group 1: Chinese herbal compound prescriptions twice a day plus Sanqi (*Panax notoginseng*) powder twice a day for 4 weeks; (3) test group 2: Chinese herbal compound prescriptions twice a day plus Sanqi (*Panax notoginseng*) powder and Zhebeimu (*Fritillaria thunbergii Miq*.) powder twice a day for 4 weeks. The primary endpoint (improvement of gastric mucosal erosion; improvement of gastric mucosal pathology) and secondary endpoints (improvement of clinical symptoms scores; improvement of the patient-reported outcome [PRO] instrument for chronic gastrointestinal diseases) were assessed using endoscopy at week 4 following the treatment. Drug-related adverse events (AEs) and adverse drug reactions (ADRs) were also compared.

**Results:**

The final analysis included 202 patients (control group, 63; test group 1, 69; test group 2, 70). At week 4, using within-group comparison, gastric mucosal erosion improved in each group following treatment with a significant difference (*P* < 0.05); there were no statistically significant differences in gastric mucosal erosion scores among the groups after treatment (*P* > 0.05); in terms of improvement of gastric mucosal erosion, the efficacy rate of the control group was 69.12%, the efficacy rate of the test group 1 was 73.24%, and the efficacy rate of the test group 2 was 69.01% and efficacy rate among the groups was not statistically significant (*P* > 0.05). As determined by acute inflammation, chronic inflammation, atrophy, intestinal metaplasia, and dysplasia, the pathological score (total score and the highest score) did not differ statistically among groups following treatment (*P* > 0.05); within the control group, the total scores of acute inflammation, chronic inflammation, atrophy, and intestinal metaplasia were significantly decreased (*P* < 0.05), but there was no significant improvement in dysplasia (*P* > 0.05); in the test group 1, chronic inflammation, atrophy, and intestinal metaplasia and dysplasia scores were significantly decreased (*P* < 0.05), but acute inflammation did not improve (*P* > 0.05); there was a significant reduction in the atrophy score in test group 2 (*P* < 0.05), but no improvement in the scores of acute inflammation, chronic inflammation, intestinal metaplasia, and dysplasia was observed (*P* > 0.05). Similarly, within the control group, the highest scores of acute inflammation, chronic inflammation, atrophy, and intestinal metaplasia were significantly decreased (*P* < 0.05), but there was no significant improvement in dysplasia (*P* > 0.05); there was a significant reduction in highest scores of atrophy, intestinal metaplasia, and dysplasia (*P* < 0.05) in test group 1, but the highest scores didn’t not improve with acute inflammation and chronic inflammation (*P* > 0.05); there was a significant reduction in the highest atrophy score in test group 2 (*P* < 0.05), but no improvement in the highest scores of acute inflammation, chronic inflammation, intestinal metaplasia, and dysplasia was observed (*P* > 0.05). Compared to the control group, the main symptom scores and total symptom scores in the test groups were lower following treatment, with a statistically significant difference (*P* < 0.05); the analysis of covariance with center, erosion type, and group as factors was applied, and the comparison among the groups in dyspepsia, defecation, and total PRO instrument scores were statistically significant (*P* < 0.05). In the study period, AEs were reported in 3 (4.23%) patients in the test group 1 and 3 (4.41%) patients in the control group; ADRs were confirmed in 3 (4.23%) patients from the test group 1 and 2 (2.94%) from the control group. AEs and ADRs were not statistically significantly different among groups (AE, *P* = 0.2213; ADR, *P* = 0.2872). No serious AE or ADR was reported.

**Conclusions:**

This study has shown that both aluminum plus magnesium suspension and Chinese herbal compound prescriptions together with *Panax notoginseng* powder are capable of improving gastric mucosal erosion and reducing gastric mucosal pathological scores, and there were no statistically significant differences among the groups in primary endpoints, indicating that Chinese herbal therapy can achieve similar efficacy than antacids in terms of primary outcomes. The aluminum plus magnesium suspension is comparable to Chinese herbal therapy in improving atrophy and intestinal metaplasia, and is inferior to Chinese herbal therapy in improving dysplasia. In addition, the Chinese herbal therapy significantly outperforms the aluminum plus magnesium suspension in improving symptoms. Therefore, the overall clinical outcome of Chinese herbal compound prescriptions together with *Panax notoginseng* powder based on TCM syndrome patterns in the treatment of erosive gastritis is superior to that of antacids.

*Trial registration* ChiCTR, ChiCTR-IPR-15005905. Registered 22 January 2015, https://www.chictr.org.cn/showproj.aspx?proj=10359

**Supplementary Information:**

The online version contains supplementary material available at 10.1186/s13020-022-00692-7.

## Background

Gastritis is defined as the histologically confirmed inflammation of the gastric mucosa and affects approximately 50% of the world’s population [1]. A variety of factors can lead to gastritis, including *Helicobacter pylori* (*H. pylori*) infection, biliary reflux into the stomach, the use of non-steroidal anti-inflammatory drugs, unbalanced diets, alcohol and acid exposure, and long-term stress [2]. Inflammation of the gastric mucosa may cause mucosal erosions, blood oozing, hyperemia (redness), and edema with inflammatory cellular infiltration of the gastric tissues [3–5]. Particularly, erosion is observed during severe exacerbations of acute gastritis and chronic gastritis [6].

In 2014, a cross-sectional survey was conducted in China involving a total of 8,892 patients recruited from 33 centers who had upper gastrointestinal symptoms and chronic gastritis confirmed by gastroscopy. Among specific types of chronic gastritis, chronic non-atrophic gastritis accounted for 49.4% and chronic gastritis with erosions accounted for 42.3% [7].

Chronic erosive gastritis is characterized endoscopically by multiple small flat or tiny elevated lesions without or with central depressions or erosions surrounding the antrum [8]. Gastric acid was considered the primary factor in causing this condition [9, 10], and, more recently, antral erosion predicted hyperchlorhydria in those with *H. pylori* negative gastritis [11]. Thus, as stated in “*Chinese consensus on chronic gastritis (2017, Shanghai)*” [12], antacids and acid-suppressing medications are recommended in order to heal mucosal erosion in the stomach and eliminate associated symptoms.

Proton pump inhibitors (PPIs), in particular, are relatively safe and effective medications. However, the limitations of these medications include a slow onset of action due to prodrugs’ mechanical limitations, an impaired inhibitory effect on gastric acid secretion following a meal, and difficulties managing nocturnal acid reflux [13]. As well, PPIs are associated with several adverse effects in the stomach, including oxyntic cell and enterochromaffin-like cell hyperplasia, as well as the occurrence of hyperplastic and fundic gland polyps as a result of hypergastrinemia [14–16]. There has also been some evidence that long-term PPI use might lead to the progression of atrophic gastritis and the development of gastric cancer [17, 18].

Since ancient times, traditional Chinese medicine (TCM), based on the theory of holism and the time-honored principle of differentiation of syndrome, has been widely used to treat digestive disorders while providing satisfying effectiveness and a cost-effectiveness. The four diagnostic elements of TCM encompass a wide range of symptoms and signs. Symptoms, physical signs, tongue appearance, and pulse reading are collected systematically as part of the syndrome differentiation process and are considered to be contributing factors to disease pathogenesis. Considering the information that is present, TCM practitioners may be able to determine the potential link between the pathologic nature and the current disease stage.

There has been growing literature demonstrating the efficacy of TCM therapies in treating gastritis [19–22]. Yet, few studies have been conducted on TCM treatments for chronic erosive gastritis, especially those interventions that corresponded to TCM syndromes. Hence, a multi-center randomized controlled trial has been conducted to assess the efficacy of Chinese herbal compound prescriptions based on TCM syndrome differentiation to provide evidence for clinical practice.

## Methods

### Ethics approval and trial registration

The present study was approved by the Ethic Committee of Xiyuan Hospital of China Academy of Chinese Medical Sciences (Ethical Approval Number: 2014XL091-2). This trial was conducted following the principles of good clinical practice and the Declaration of Helsinki guidelines. The informed consent of all subjects was obtained at the time of enrollment. This was a randomized, standard clinical trial (Chinese ClinicalTrials.gov: ChiCTR-IPR-15005905).

### Study population

This was a multi-center, randomized, positive drug control clinical trial, to evaluate the safety and efficacy of Chinese herbal compound prescriptions in treating chronic erosive gastritis with eight TCM syndromes. Patients were recruited from the following 6 Chinese centers: Xiyuan hospital, China Academy of Chinese Medical Sciences (Beijing), Kunshan Traditional Chinese Medicine Hospital (Kunshan), The Hospital of Shanxi University of Chinese Medicine (Taiyuan), Guangdong Provincial Hospital of Chinese Medicine (Guangzhou), Wuhan No.1 Hospital (Wuhan Integrated TCM and Western Medicine Hospital) (Wuhan), and Shenzhen Traditional Chinese Medicine Hospital (Shenzhen).

### Diagnostic criteria

#### Diagnostic criteria of western medicine

“*The Consensus on Chronic Gastritis in China in 2012*” published by Members of Chinese Society of Gastroenterology, was referenced in diagnosing gastritis [23].

1. Endoscopic diagnosis: atrophic gastritis is defined as a condition in which the color of the mucosa alternates between red and white, predominating with white, the rugae flatten or disappear, the blood vessels are visible, and the mucosa appears granular or nodular.
2. Erosion: erosion can be categorized as either flat or bulge-shaped. Single or multiple erosive lesions of the gastric mucosa may be present in the former; the size of the lesions can vary from the size of the needle tip to a few centimeters in diameter. The latter can be characterized by a single or several verrucous lesions, enlarged folds or plump-like bulges, ranging in diameter from 5 to 10 mm, with mucosal defects or umbilical depressions on top and erosion in the center.
3. Erythema: erythema is distinguished from its surrounding mucosa by a marked reddening.
4. Intramucosal bleeding: there are several types of intramucosal bleeding, including spotty hemorrhaging, patchy bleeding, and non-raised red or dark red hemorrhagic spots (with or without oozing of blood; fresh or old).
5. Pathological diagnosis: Pathologically, chronic gastritis with inherent gland atrophy, as shown by biopsy, can be diagnosed as atrophic gastritis, irrespective of the number of biopsy specimens and the degree of atrophy.
6. Gastrointestinal symptoms: the main clinical symptoms include epigastric pain and epigastric bloating while the secondary symptoms include early satiety, postprandial fullness and bloating, and epigastric burning.

#### Diagnostic criteria of TCM

For TCM syndromes, “*TCM Consensus on Chronic Atrophic Gastritis Diagnosis and Treatment (2009, Shenzhen)*” was used, including syndrome of stagnation of liver-qi, syndrome of stagnant heat in liver and stomach, syndrome of spleen-stomach dampness-heat, syndrome of spleen-deficiency and qi-stagnation, syndrome of spleen and stomach deficiency-cold, syndrome of stomach-yin deficiency, syndrome of liver-stomach-yin deficiency, and syndrome of deficiency of qi and yin [24].

### Inclusion criteria

A total of 216 patients with chronic atrophic gastritis with erosions were enrolled and divided into three groups: test group 1, test group 2, and control group, with 72 patients in each group.

Inclusion criteria were as follows: (1) participants who met the diagnostic criteria for chronic atrophic gastritis and had predominance of gastric antral atrophy; (2) patients aged 18 to 70 years; (3) patients with a gastric erosion grade 2 (3–5 erosions) or above, and gastric antral erosion is present; (4) patients with at least one of the symptoms including early satiety, postprandial fullness and bloating, epigastric pain, and epigastric burning; and (5) patients who are able and willing to provide informed consent and to attend treatment.

### Exclusion criteria

The exclusion criteria were as follows: (1) patients with *H. pylori* infection; (2) patients with type A gastritis, acute erosive gastritis, atrophic gastritis with severe dysplasia, upper gastrointestinal hemorrhage, peptic ulcer, or neoplasms of the gastrointestinal tract; (3) patients with evidence of gastrointestinal organic lesions such as pancreatitis and liver cirrhosis or those with diseases that can lead to disordered gastrointestinal motility, including hyperthyroidism, diabetes, chronic renal failure, psychiatric illness, or neurological lesions; (4) patients with significant impairments in the cardiac, hepatic, renal, hematologic, and hematopoietic systems, and tumors; (5) patients who continue any medications that may affect the function of the digestive tract, including antacids, H_2_-receptor antagonists, PPIs, gastrin receptor antagonists, anticholinergic drugs, prokinetics, prostaglandin analogs, or TCM therapies that have similar effects; (6) patients who had a history of alcohol drinking within 1 week; (8) pregnant and lactating women; (9) patients with known hypersensitivity to components from drugs used in this study; and (10) patients who are participating in other clinical trials.

### Rejection criteria

The rejection criteria were as follows: (1) patients who are misdiagnosed or erroneously included; (2) patients who met exclusion criteria; (3) patients who did not take the drugs or were not observed for at least one time point; and (4) patients who take forbidden drugs so that efficacy cannot be determined.

### Drop-out criteria

Reasons for drop-out are: (1) patients who drop out of the trial by themselves; (2) patients who are lost in the follow-up period; (3) patients who have poor compliance that affected the evaluation of efficacy and safety; and (4) patients who are withdrawn from the trial at any time by the treating physician due to other disease.

### Discontinuation criteria

Discontinuation criteria were as follows: (1) patients who have clinically relevant safety concerns and serious adverse events; (2) the drugs which are insufficiently effective for further trials to be conducted; (3) major errors in clinical research protocol; and (4) administrative decision to withdraw the trial.

### Randomization and intervention

#### Randomization

Participants will be randomized at a 2:1 ratio by the Central Randomization System of the Good Clinical Practice Unit of Xiyuan Hospital, which is an online, central randomized service. Using the patients’ dates of birth and erosion types, the investigators applied for the randomization code in the randomization system, and the randomization system emailed the investigators the results.

#### Interventions

Patients in the control group received aluminum plus magnesium suspension (15 ml/sachet; produced by Yangzhou Yiyang Pharmaceutical Co., Ltd.) thrice per day, 1 h after meals, 1 sachet at a time for 4 weeks.

Participants in test group 1 received Chinese herbal compound prescriptions plus *Sanqi* (*Panax notoginseng*) powder.

Participants in test group 2 received Chinese herbal compound prescriptions plus *Sanqi* (*Panax notoginseng*) powder and *Zhebeimu* (*Fritillaria thunbergii* Miq.) powder.

Chinese herbal compound prescriptions were consistent with TCM syndromes; the prescription was administered orally 1 h after meals, twice a day for 4 weeks; preprandial administration was allowed if there was a pre-meal hunger.

Powder (manufactured by Beijing Huamiao Pharmaceutical Co., Ltd.) was taken orally each day, before lunch and dinner, 3 g once, twice a day for 4 weeks. Specifically, *Sanqi* (*Panax notoginseng*) powder was administered orally at 3 g once in test group 1; *Sanqi* (*Panax notoginseng*) powder and *Zhebeimu* (*Fritillaria thunbergii* Miq.) powder were administered orally at 1.5 g once, respectively.

The TCM treatments were given as follows:

1. Participants of syndrome of stagnation of liver-qi received *Chaihu* (*Radix bupleuri*)—9 g, *Baishao* (*Paeoniae* Radix *Alba*)- 15 g, *Zhiqiao* (*Fructus aurantii*)—12 g, *Xiangfu* (*Cyperus rhizome*)—12 g, *Chenpi* (*Citrus reticulata*)—9 g, *Qingpi* (*Citri Reticulatae Pericarpium Viride*)—9 g, *Xiangyuanpi* (*Citrus medica*)—12 g, *Foshou* (*Citrus medica* var. *Sarcodactylis*)—12 g, *Chuanxiong* (*Ligusticum chuanxiong Hort*)—9 g, *Yanhusuo* (*Corydalis yanhusuo W.T. Wang*)—12 g, *Sharen* (*Amomum villosum Lour.*)—6 g, and *Shenggancao* (*Glycyrrhiza uralensis* Fisch.)—6 g;
2. Participants of syndrome of stagnant heat in liver and stomach received *Zisu* (*Perilla frutescens* L. Britt.)—12 g, *Xiangfu* (*Cyperus rhizome*)—12 g, *Chenpi* (*Citrus reticulata*)—10 g, *Zhishi* (*Citrus aurantium* L.)—12 g, *Sharen* (*Amomum villosum Lour.*)—6 g, *Longdan* (*Gentiana scabra Bunge*)—6 g, *Huanglian* (*Coptis chinensis* Franch.)—6 g, *Wuzhuyu* (*Evodiae Fructus*)—2 g, *Wuzeigu* (*Sepiella maindroni de Rochebrune*)—30 g, *Pugongying* (*Taraxacum* mongolicum Hand.-Mazz.)—18 g, and *Shenggancao* (*Glycyrrhiza uralensis* Fisch.)—6 g;
3. Participants of syndrome of spleen-stomach dampness-heat received *Dangshen* [*Codonopsis pilosula* (Franch.) Nannf.]—15 g, *Cangzhu* [*Atractylodes lancea* (Thunb.) DC.]—15 g, *Banxia* [*Pinellia ternata* (Thunb) Breit.]—9 g, *Chenpi* (*Citrus reticulata*)—12 g, *Huangqin* (*Scutellaria baicalensis* Georgi)—12 g, *Peilan* (*Eupatorium fortunei* Turcz.)—15 g, *Zhishi* (*Fructus aurantii*)—12 g, *Sharen* (*Amomum villosum Lour.*)—6 g, *Baikouren* (*Amomum kravanh* Pierre ex Gagnep.)—6 g, *Huashi* (talcum)—10 g, *Zhebeimu* (*Fritillaria thunbergii* Miq.)—15 g, and *Zhigancao* (*Radix Glycyrrhizae preparata*)—6 g;
4. Participants of syndrome of spleen-deficiency and qi-stagnation received *Huangqi* (*Astragali Radix*)—18 g, *Dangshen* [*Codonopsis pilosula* (Franch.) Nannf.]—15 g, *Baizhu* (*Atractylodes macrocephala* Koidz.)—18 g, *Fuling* [*Wolfiporia cocos* (F.A. Wolf) Ryvarden & Gilb.]—18 g, *Banxia* [*Pinellia ternata* (Thunb) Breit.]—9 g, *Muxiang* (*Radix Aucklandiae*)—12 g, *Zhiqiao* (*Fructus aurantii*)—15 g, *Sharen* (*Amomum villosum Lour.*)—9 g, *Shenqu* (*Massa Medicata Fermentata*)—15 g, *Yanhusuo* (*Corydalis yanhusuo W.T. Wang*)—12 g, and *Shenggancao* (*Glycyrrhiza uralensis* Fisch.)—6 g;
5. Participants of syndrome of spleen and stomach deficiency-cold received *Zhihuangqi* (*Radix Astragali* preparata)—18 g, *Dangshen* [*Codonopsis pilosula* (Franch.) Nannf.]—15 g, *Chaobaizhu* (*Atractylodes macrocephala* Koidz.)—15 g, *Baishao* (*Paeoniae* Radix *Alba*)—15 g, *Fuling* [*Wolfiporia cocos* (F.A. Wolf) Ryvarden & Gilb.]—18 g, *Banxia* [*Pinellia ternata* (Thunb) Breit.]—9 g, *Zhiqiao* (*Fructus aurantii*)—12 g, *Muxiang* (*Radix Aucklandiae*)—12 g, *Chenpi* (*Citrus reticulata*)—12 g, *Ganjiang* (*Zingiberis Rhizoma*)—9 g, and *Shenggancao* (*Glycyrrhiza uralensis* Fisch.)—6 g;
6. Participants of syndrome of stomach-yin deficiency received *Beishashen* (*Radix Glehniae*)—15 g, *Maidong* [*Ophiopogon japonicus* (Linn. f.) Ker-Gawl.]—15 g, *Baihe* (*Lilium brownii* F.E.Brown var. *viridulum* Baker)—30 g, *Wuyao* [*Lindera* aggregata (Sims) Kosterm]—12 g, *Shihu* (*Dendrobium nobile* Lindl)—12 g, *Chaobaizhu* (*Atractylodes macrocephala* Koidz.)—15 g, *Zhiqiao* (*Fructus aurantii*)—12 g, *Foshou* (*Citrus medica* var. *Sarcodactylis*)- 15 g, *Sharen* (*Amomum villosum Lour.*)—6 g, *Walengzi* (*Arcae Concha*)—30 g, *Baishao* (*Paeoniae* Radix *Alba*)—15 g, *Chaoguya* (*Setariae Fructus Germinatus*)—15 g, *Chaomaiya* (*Hordei Fructus Germinatus*)—15 g, and *Shenggancao* (*Glycyrrhiza uralensis* Fisch.)—6 g;
7. Participants of syndrome of liver-stomach-yin deficiency received *Chaihu* (*Radix bupleuri*)—9 g, *Baishao* (*Paeoniae* Radix *Alba*)—15 g, *Danggui* (Angelicae Sinensis Radix)—12 g, *Goujizi* (*Lycium barbarum L.*)—18 g, *Beishashen* (*Radix Glehniae*)—15 g, *Maidong* [*Ophiopogon japonicus* (Linn. f.) Ker-Gawl.]—15 g, *Baizhu* (*Atractylodes macrocephala* Koidz.)—15 g, *Zhiqiao* (*Fructus aurantii*)—12 g, *Sharen* (*Amomum villosum Lour.*)—6 g, *Xiangyuanpi* (*Citrus medica*)—12 g, *Foshou* (*Citrus medica* var. *Sarcodactylis*)—15 g, *Wuzeigu* (*Sepiella maindroni de Rochebrune*)—30 g, and *Shenggancao* (*Glycyrrhiza uralensis* Fisch.)—6 g;
8. Participants of syndrome of deficiency of qi and yin received *Huangqi* (*Astragali Radix*)—15 g, *Beishashen* (*Radix Glehniae*)—15 g, *Maidong* [*Ophiopogon japonicus* (Linn. f.) Ker-Gawl.]—15 g, *Shihu* (*Dendrobium nobile* Lindl)—12 g, *Baizhu* (*Atractylodes macrocephala* Koidz.)—15 g, *Fuling* [*Wolfiporia cocos* (F.A. Wolf) Ryvarden & Gilb.]—15 g, *Zhiqiao* (*Fructus aurantii*)—12 g, *Muxiang* (*Radix Aucklandiae*)—12 g, *Sharen* (*Amomum villosum Lour.*)—9 g, *Shenqu* (*Massa Medicata Fermentata*)—15 g, and *Shenggancao* (*Glycyrrhiza uralensis* Fisch.)—6 g.

### Observation indicators

#### Primary clinical outcome indicators

1. The improvement in gastric mucosal erosion observed by endoscopy following the treatment: each patient underwent an endoscopy at baseline and 4 weeks after treatment initiation. The lesions in the gastric antrum, fundus and body were analyzed altogether. “Cure”, “markedly effective” and “effective” were merged to calculate efficacy rate. The endoscopy results after treatment were assessed as follows: cure (disappearance of erosions), markedly effective (the erosion score decreased by at least two points), effective (the erosion score has decreased by one point) and ineffective (no change or worse) [25];
2. Changes in the pathological score of gastric mucosa (acute inflammation, chronic inflammation, atrophy, intestinal metaplasia, dysplasia) following the treatment: the total and highest pathological scores of the gastric antrum, fundus and body were compared for each group. The reduction in the aforementioned pathological scores was used to determine the pathological efficacy rate of gastric mucosa.

Throughout the study, the same expert endoscopist and experienced gastrointestinal pathologist performed all endoscopic procedures and pathologic examinations in each center. All endoscopists and pathologists were uniformly trained and qualified in a standardized manner.

#### Secondary outcome indicators

1. Clinical symptom scores [25];
2. The patient-reported outcome (PRO) instrument for chronic gastrointestinal diseases [19, 26]; (3) Safety assessments that included adverse events (AEs) and adverse drug reactions (ADRs), including any abnormalities in physical examination, blood, urine and stool routine test and electrocardiogram, liver and renal function.

### Sample size estimation

The design of the study is a randomized, controlled, multi-center, clinical trial. The previous clinical literature [27–29] showed that the efficacy rate was 75% in the aluminum plus magnesium suspension group and 91% in the Chinese herbal compound prescriptions plus powder group.

The sample size was calculated by using a clinical research sample size calculator (CRESS V1.3) [30] with the formula of sample size for rate below (Eqs. 1 and 2).

1 $$ n_{c} = \frac{{\left[ {\mu_{1 - \alpha } \sqrt {\overline{p}(1 - \overline{p}(1 + 1/k)} + \mu_{1 - \beta } \sqrt {p_{T} (1 - p_{T} /k + p_{c} (1 - p_{c} } )} \right]^{2} }}{{[(p_{T} - p_{c} - \Delta ]^{2} }} $$

2 $$ n_{t} = kn_{c} $$

Based on these results, in this study, we set, a β of 0.20 (or 20%), with a one-sided α level of 0.025; the sample size was calculated as 120 cases and 60 cases in the test group and control group, respectively at a ratio of 2:1. With the dropout rate controlled within 20%, we concluded that a total of 216 patients with 144 for the test group and 72 for the control group would need to be recruited to ensure statistically significant results.

### Statistical analyses

All analyses were conducted with SAS software 9.4 (SAS Institute, Cary, NC, USA). (1) Measurement data were analyzed by a t test, paired t test, rank-sum test, paired rank sum test, and median test. (2) Numerical data were analyzed by the χ^2^ test and Fisher exact test; ranked data were analyzed using a rank-sum test and the Cochran-Mantel–Haenszel (CMH) test. (3) Efficacy measures were strictly conducted according to the full analysis set (FAS); the CMH test was conducted for multi-center numerical data; measurement data were compared using analysis of variance (ANOVA) or rank-sum test depending on data distribution. To confounding factors that were difficult to the control prior to the treatment or without control, least-square means (LSM) and their 95% confidence intervals (CI) for results from analysis of covariance (ANCOVA) or logistic regression analysis was adopted if there was baseline imbalance between the groups. (4) Variability test was used; test level of α = 0.05, and *P* values less than 0.05 were considered statistically significant.

## Results

### Subjects enrollment

**Fig. 1:**
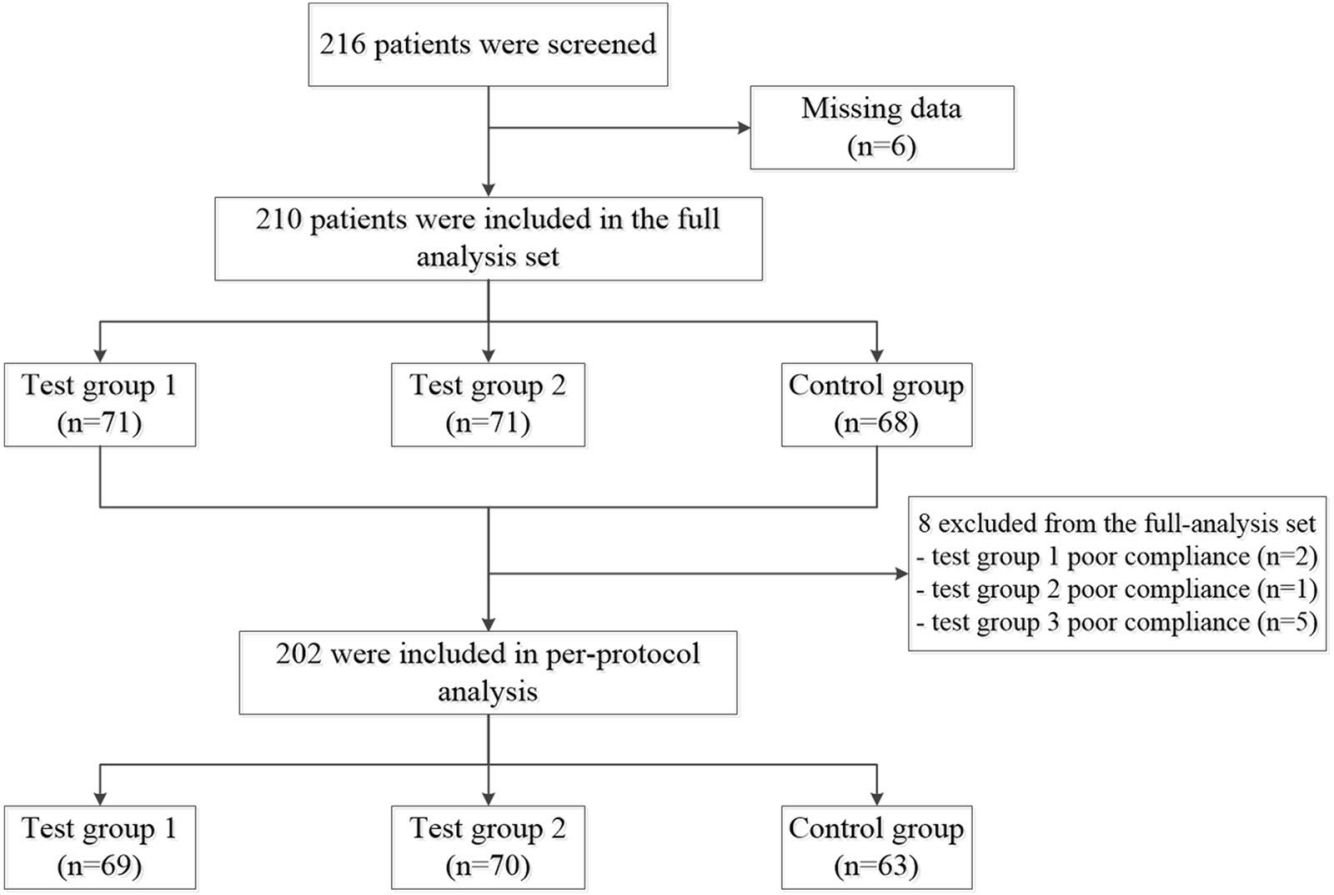
Flowchart of the patients included in the study

From November 2015 through May 2018, 216 subjects were enrolled in this study, of which 210 were randomly assigned to either test group 1 (n = 71), test group 2 (n = 71), or control group (n = 68). Eight patients were excluded prior to performing the per-protocol (PP) analysis due to poor compliance. Consequently, data from 202 patients were used for the PP analysis. Figure 1 represents a detailed flowchart of this study.

### Demographic characteristics

Table 1 shows patients’ demographic characteristics. There were no differences among the groups in terms of age, sex, ethnicity, marital status, education, and occupation (*P* > 0.05).

**Table 1:** Baseline demographic characteristics of the study patients

Characteristics	Control group (n = 68)	Test group 1 (n = 71)	Test group 2 (n = 71)	*P* value	statistical analysis
Age, yr				0.8423	ANOVA
Mean ± SD	53.81 ± 8.65	54.58 ± 9.97	53.73 ± 9.73		
Maximum, minimum	30,68	22,72	22,69		
Median	55	57	54		
95% CI	(51.71 ~ 55.9)	(52.22 ~ 56.94)	(51.43 ~ 56.03)		
Sex				0.8397	χ^2^
Men	35 (51.47)	39 (54.93)	40 (56.34)		
Women	33 (48.53)	32 (45.07)	31 (43.66)		
Ethnicity				0.5967	χ^2^
Han	67 (98.53)	70 (98.59)	71 (100)		
Non-Han	1 (1.47)	1 (1.41)	0 (0)		
Marital status				0.4103	χ^2^
Unmarried	0 (0)	1 (1.41)	2 (2.82)		
Married	66 (97.06)	70 (98.59)	69 (97.18)		
Divorced	1 (1.47)	0 (0)	0 (0)		
Widowed	1 (1.47)	0 (0)	0 (0)		
Education				0.2776	χ^2^
Illiteracy (< 1 yr)	2 (2.94)	0 (0)	3 (4.23)		
Elementary school (1–6 yr)	12 (17.65)	24 (33.8)	21 (29.58)		
Junior high school (7–9 yr)	14 (20.59)	16 (22.54)	19 (26.76)		
High school or technical secondary school (10–12 yr)	26 (38.24)	20 (28.17)	14 (19.72)		
Junior college and college or above (≥ 13 yr)	10 (14.71)	9 (12.68)	11 (15.49)		
Unknown	4 (5.88)	2 (2.82)	3 (4.23)		
Occupation				0.0289	χ^2^
Mental labor	32 (47.76)	23 (32.39)	21 (29.58)		
Physical labor	20 (29.85)	32 (45.07)	40 (56.34)		
Other	15 (22.39)	16 (22.54)	10 (14.08)		

All values are presented as the mean ± standard deviation (SD) or number (%)

### Erosion type and distribution of syndrome types

As shown in Table 2, there was no statistically significant differences among groups when erosion type and syndrome type were compared (*P* > 0.05).

**Table 2:** Erosion types and syndrome distributions

	Control group (n = 68)	Test group 1 (n = 71)	Test group 2 (n = 71)	*P* value	Statistical analysis
Erosion type				0.7485	χ^2^
Flat	48 (33.57)	49 (34.27)	46 (32.17)		
Bulge	20 (29.85)	22 (32.84)	25 (37.31)		
Syndrome type				0.1337	χ^2^
Syndrome of stagnation of liver-qi	16 (39.02)	13 (31.71)	12 (29.27)		
Syndrome of stagnant heat in liver and stomach	12 (34.29)	10 (28.57)	13 (37.14)		
Syndrome of spleen-stomach dampness-heat	13 (28.26)	17 (36.96)	16 (34.78)		
Syndrome of spleen-deficiency and qi-stagnation	9 (17.65)	20 (39.22)	22 (43.14)		
Syndrome of spleen and stomach deficiency-cold	1 (5)	11 (55)	8 (40)		

All values are presented as the mean ± standard deviation (SD) or number (%)

### Primary efficacy assessment

#### Improvement of gastric mucosal erosion

**Fig. 2:**
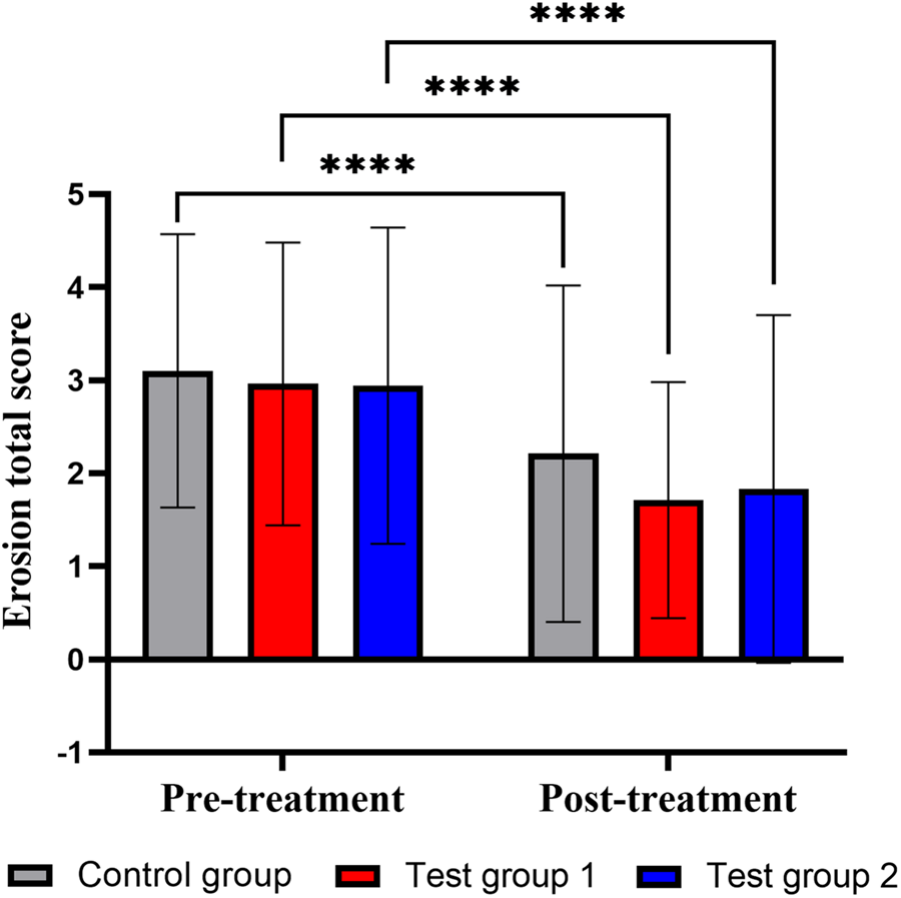
Erosion scores (total score) differences between pre- and post-treatment. All values are presented as the mean ± SD. *P < 0.05, **P < 0.01, ***P < 0.001, and ****P < 0.0001 versus post-treatment

The changes in gastric mucosal erosion of each group following treatment can be seen in Fig. 2. There were no statistical differences among groups in erosion score (total score) prior to treatment (*P* > 0.05). Using within-group comparison, gastric mucosal erosion improved in each group following treatment with a significant difference (*P* < 0.05); there were no statistically significant differences in gastric mucosal erosion scores among the groups after treatment (*P* > 0.05).

**Fig. 3:**
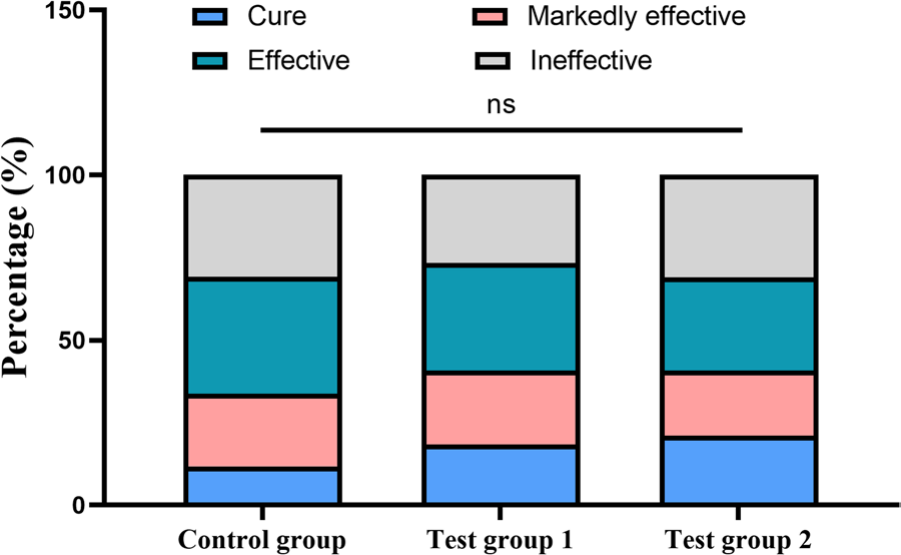
Improvement in gastric mucosal erosion and efficacy rate of treatment on gastric mucosal erosion in each group. All values are presented in percentage (%). The data were analyzed by CMH χ^2^ test. ns: non-significant

As shown in Fig. 3, the efficacy rate of the control group was 69.12%, the efficacy rate of the test group 1 was 73.24%, and the efficacy rate of the test group 2 was 69.01%; efficacy rate among the groups was not statistically significant (*P* > 0.05).

#### Improvement of gastric mucosal pathology

**Fig. 4:**
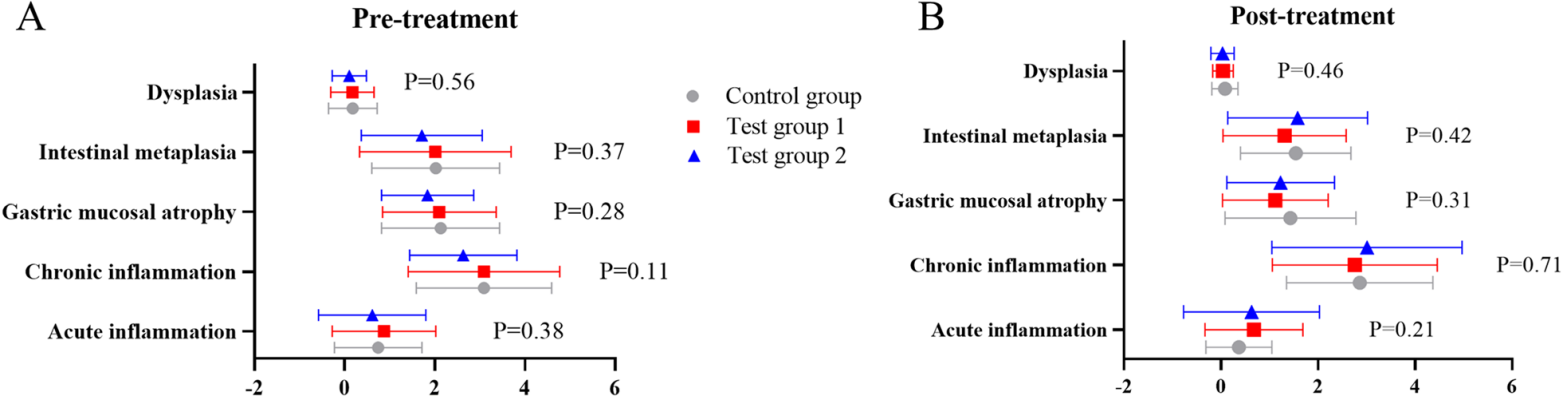
**A** Pre-treatment pathological scores in each group (total score); **B** post-treatment pathological scores in each group (total score)

As determined by acute inflammation, chronic inflammation, atrophy, intestinal metaplasia, and dysplasia, there were no statistical differences among groups in pathological score (total score) prior to treatment (P > 0.05) (Fig. 4A). The pathological score (total score) did not differ statistically among groups following treatment (*P* > 0.05) (Fig. 4B).

**Fig. 5:**
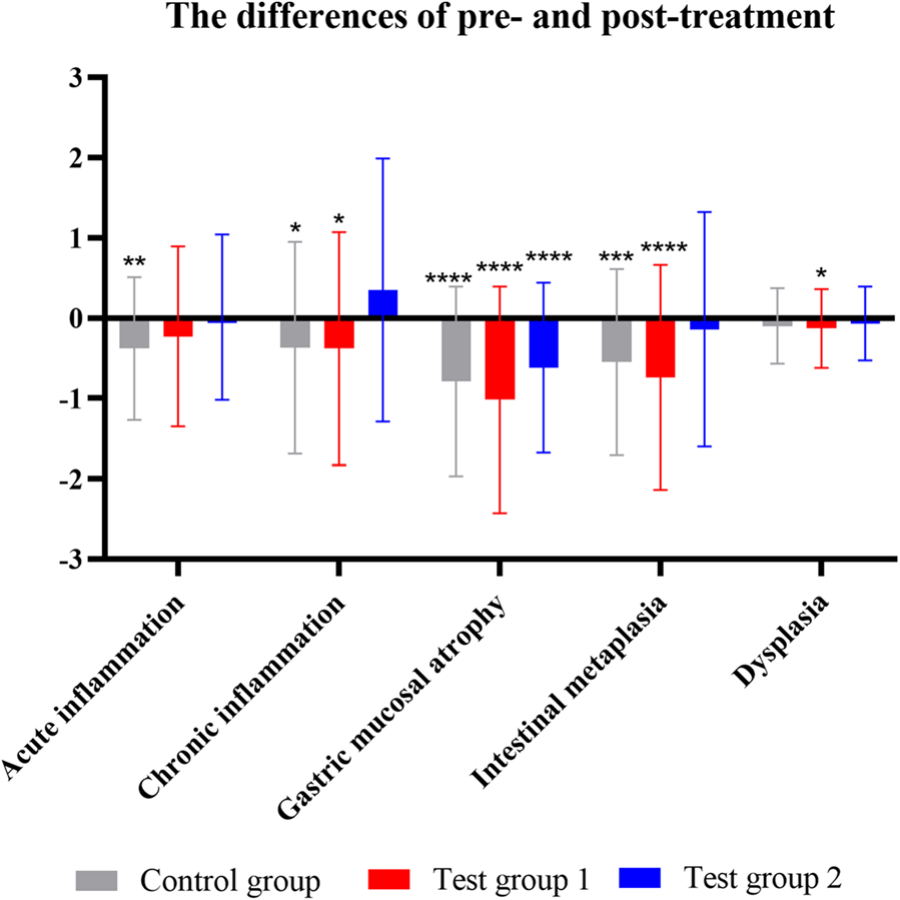
Differences of the pre- and post treatment pathological scores in each group (total score). *P < 0.05, **P < 0.01, ***P < 0.001, and **** p < 0.0001 versus post-treatment

As shown in Fig. 5, within the control group, the total scores of acute inflammation, chronic inflammation, atrophy, and intestinal metaplasia were significantly decreased (*P* < 0.05), but there was no significant improvement in dysplasia (*P* > 0.05); in the test group 1, chronic inflammation, atrophy, and intestinal metaplasia and dysplasia scores were significantly decreased (*P* < 0.05), but acute inflammation did not improve (*P* > 0.05); there was a significant reduction in the atrophy score in test group 2 (*P* < 0.05), but no improvement in the scores of acute inflammation, chronic inflammation, intestinal metaplasia, and dysplasia was observed (*P* > 0.05). The efficacy rate of pathological score (total score) did not differ statistically among groups (*P* > 0.05) (Additional file 1: Fig. S1).

**Fig. 6:**
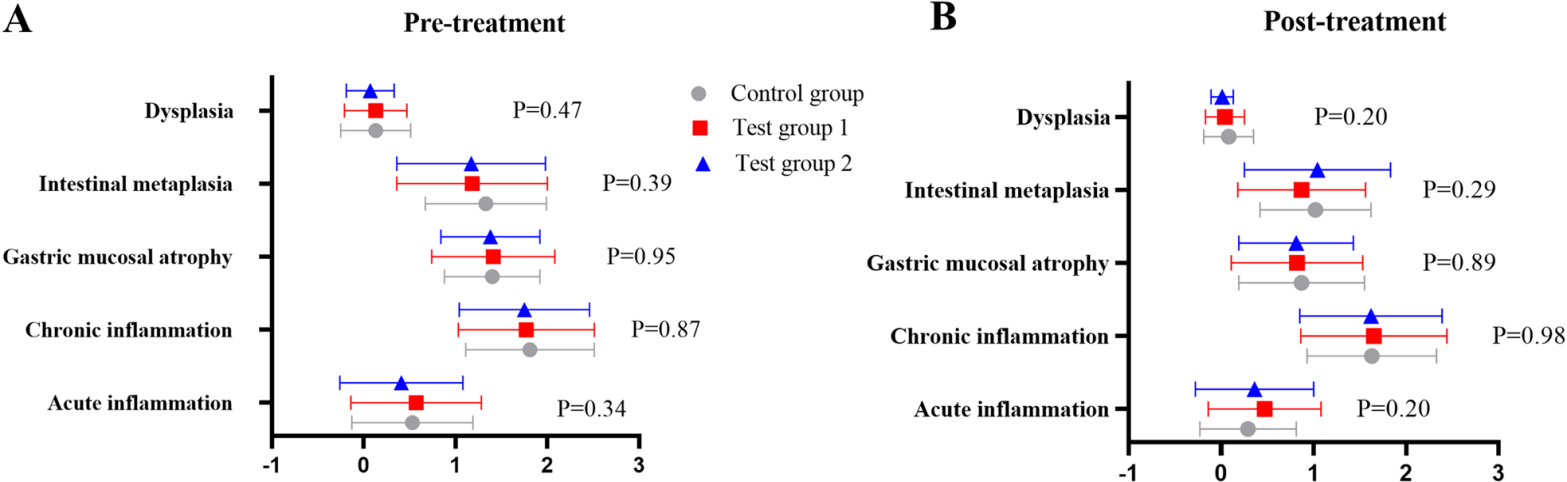
**A** Pre-treatment pathological scores (highest score) in each group; **B** post-treatment pathological scores (highest score) in each group

**Fig. 7:**
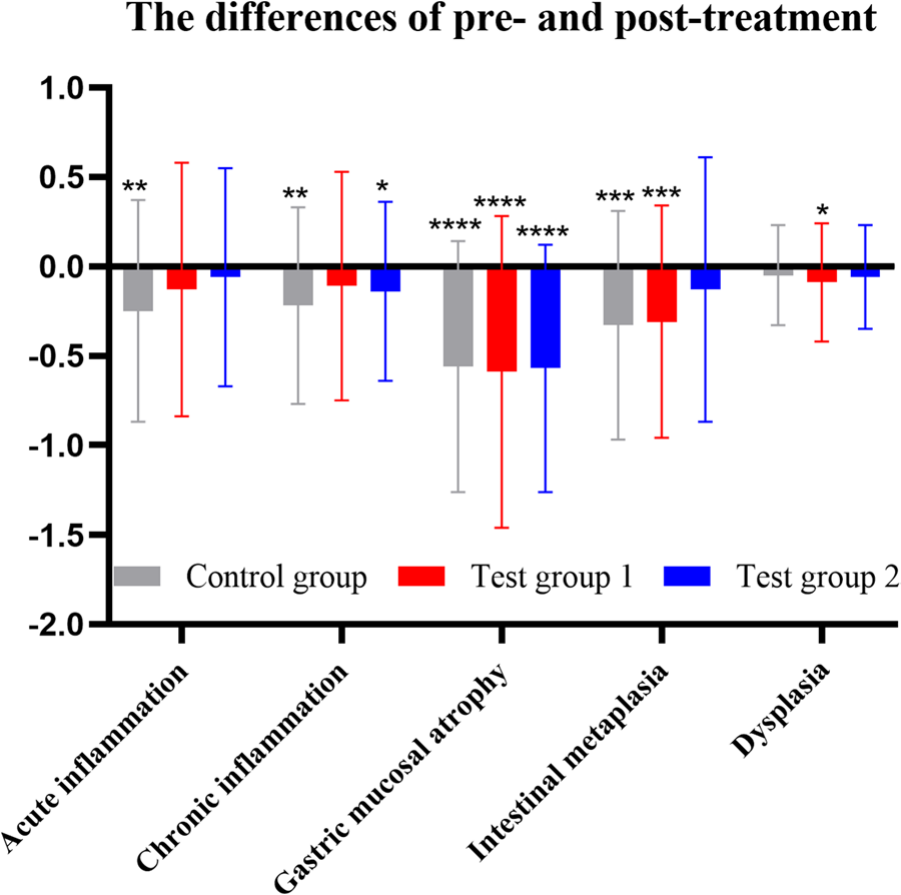
Differences of the pre- and post treatment pathological scores in each group (highest score). *P < 0.05, **P < 0.01, ***P < 0.001, and ****P < 0.0001 versus post-treatment

There were no statistical differences among groups in pathological score (highest score) prior to treatment (*P* > 0.05) (Fig. 6A). No statistically significant differences of pathological scores (highest score) were found among the groups following treatment (*P* > 0.05) (Fig. 6 B). In Fig. 7, within the control group, the scores of acute inflammation, chronic inflammation, atrophy, and intestinal metaplasia were significantly decreased (*P* < 0.05), but no significant improvement in dysplasia was found (*P* > 0.05); there was a significant reduction in scores of atrophy, intestinal metaplasia, and dysplasia (*P* < 0.05) in test group 1, but the scores didn’t not improve with acute inflammation and chronic inflammation (*P* > 0.05); there was a significant reduction in the atrophy score in test group 2 (*P* < 0.05), but no improvement in the scores of acute inflammation, chronic inflammation, intestinal metaplasia, and dysplasia was observed (*P* > 0.05). There was no significant difference in the efficacy rate of pathological score (highest score) among the groups (*P* > 0.05) (Additional file 1: Fig. S2).

### Secondary efficacy assessment

#### Improvement of clinical symptoms scores

**Fig. 8:**
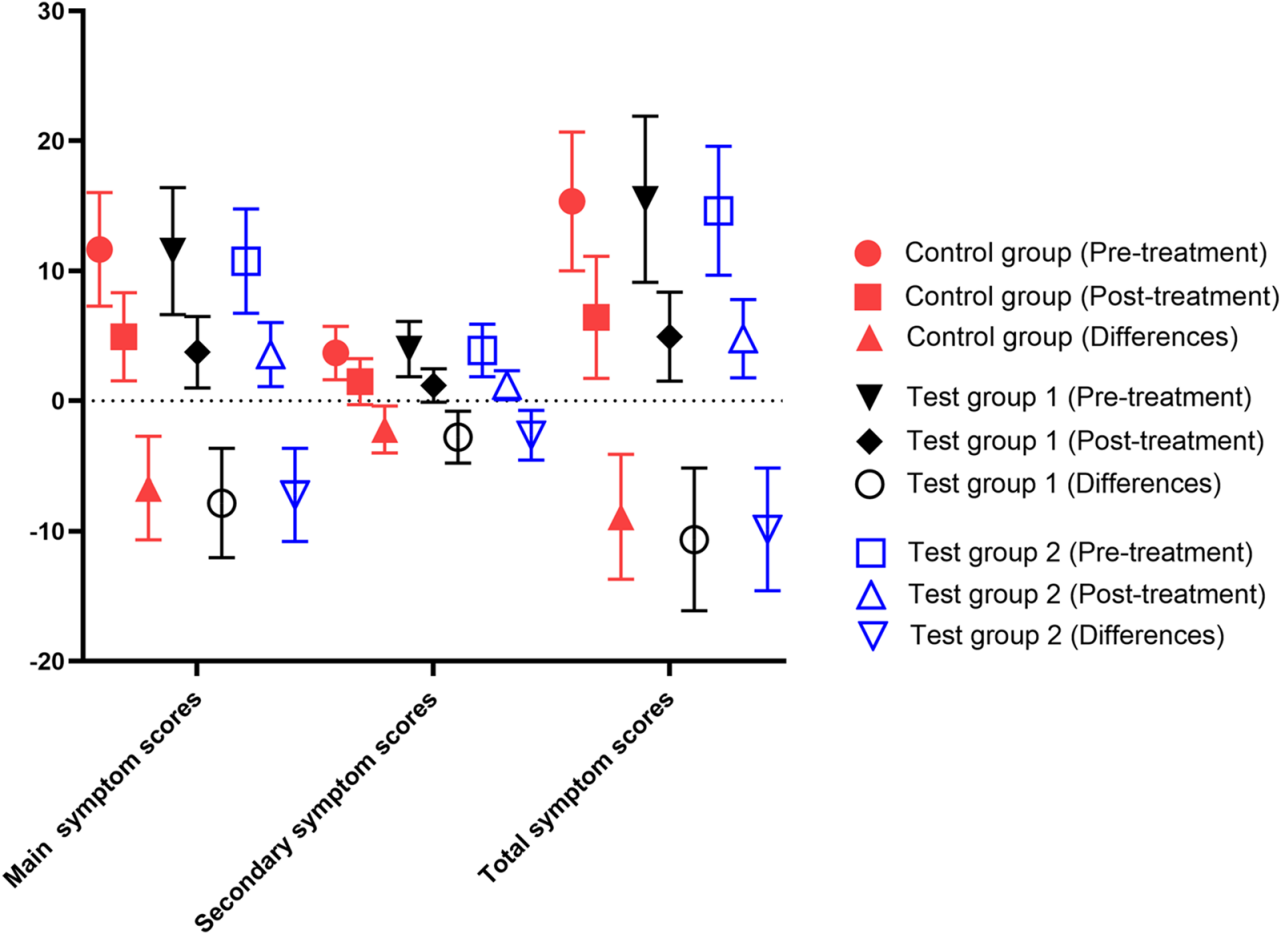
Baseline symptom scores and differences of the pre- and post treatment symptom scores in each group

As illustrated in Fig. 8, there were not statistically significant differences in baseline clinical symptoms scores among the groups (*P* > 0.05); compared to the control group, the main symptom scores and total symptom scores in the test groups were lower following treatment, with a statistically significant difference (*P* < 0.05); there was no statistically significant difference in secondary symptom improvement among the groups (*P* > 0.05). Within each group, clinical symptoms have all improved following treatment (*P* < 0.05) (Fig. 8).

#### Improvement of the PRO instrument scores

**Fig. 9:**
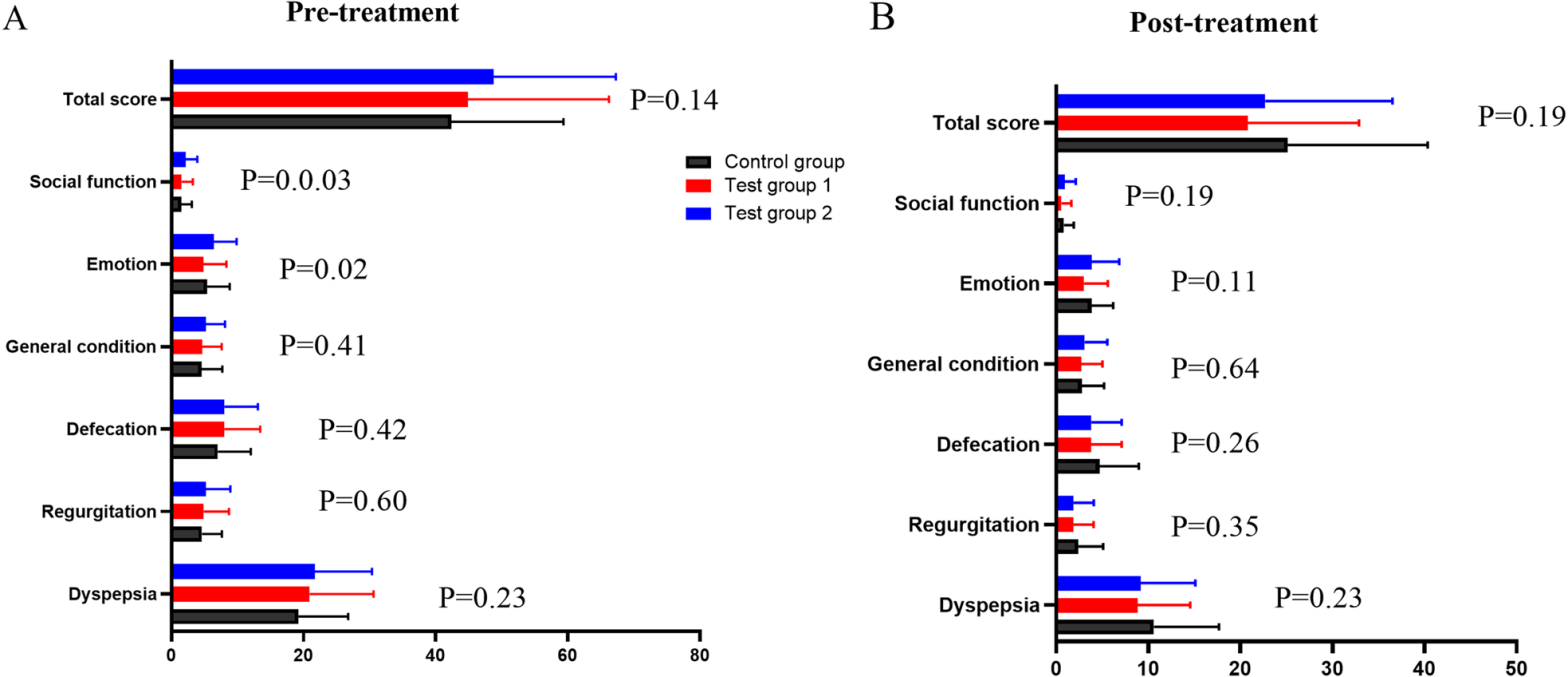
**A** Pre-treatment PRO instrument scores in each group; **B** post-treatment PRO instrument scores in each group

In terms of the PRO instrument evaluation, there were not statistically significant differences in baseline PRO instrument scores (*P* > 0.05), but there were statistically significant differences in emotion and social function (*P* < 0.05) (Fig. 9A). There were no statistically significant differences among the groups following treatment (*P* > 0.05) (Fig. 9B).

**Fig. 10:**
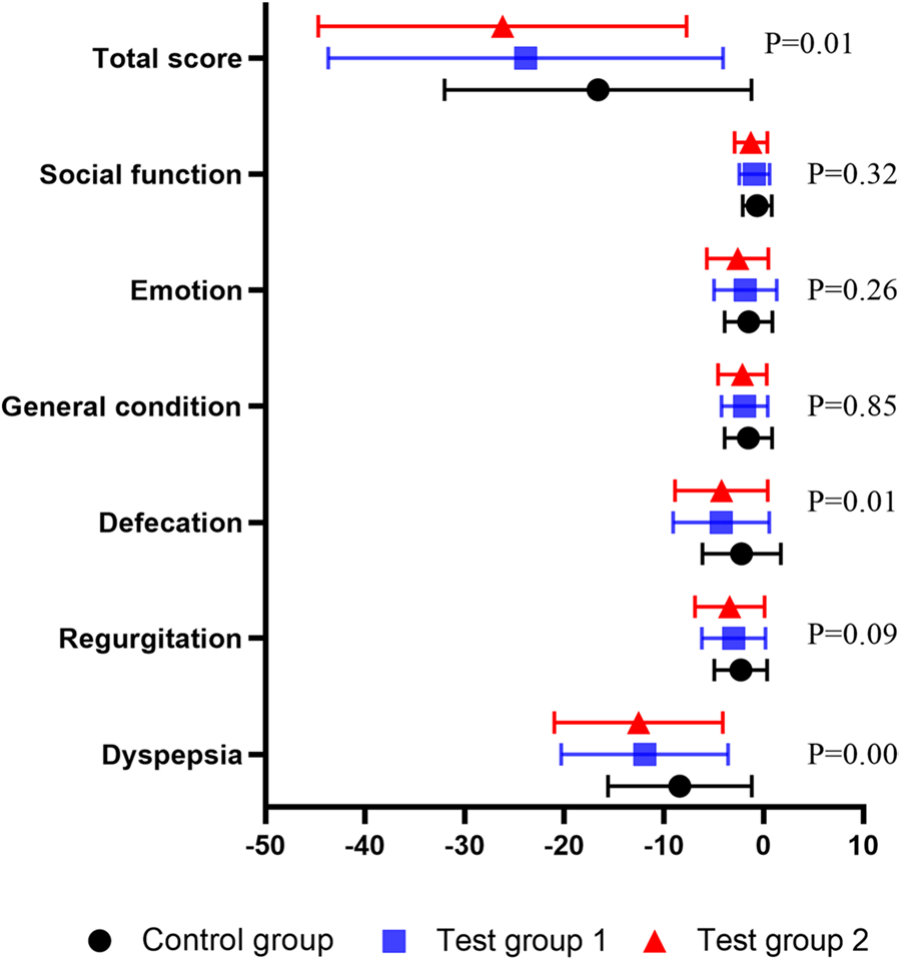
Differences of the pre- and post treatment PRO instrument scores in each group

The PRO instrument score were reduced within each group (Fig. 10). The ANCOVA with center, erosion type, and group as factors was applied, and the comparison among the groups in dyspepsia, defecation, and total PRO instrument scores were statistically significant (*P* < 0.05) (Fig. 10).

### Safety

In the study period, 3 (4.23%) patients in the test group 1 and 3 (4.41%) patients in the control group experienced AEs; ADRs were confirmed in 3 (4.23%) patients from the test group 1 and 2 (2.94%) from the control group (Table 3). Urinary tract infection (UTI) was the most common event, and AEs and ADRs were not statistically significantly different among groups (AE, *P* = 0.2213; ADR, *P* = 0.2872) (Tables 3, 4). No serious AE or ADR was reported.

**Table 3:** The incidence of adverse events and adverse drug reactions

	Test group 1 (n = 71)	Test group 2 (n = 71)	Control group (n = 68)	*P* value	Statistical analysis
	N. (%)	N. (%)	N. (%)		
AEs	3 (4.23)	0 (0)	3 (4.41)	0.2213	Fisher Exact Probability Test
ADRs	3 (4.23)	0 (0)	2 (2.94)	0.2872	Fisher Exact Probability Test

**Table 4:** The incidence of adverse events and adverse drug reactions (test groups combined for analysis)

	Test group (n = 142)	Control group (n = 68)	*P* value	Statistical analysis
	N. (%)	N. (%)		
AEs	3 (2.11)	3 (4.41)	0.3914	Fisher Exact Probability Test
ADRs	3 (2.11)	2 (2.94)	0.6597	Fisher Exact Probability Test

The combined analysis of the test groups *versus* the control group did not demonstrate any statistical significance in terms of AEs or ADRs (AE, *P* = 0.3914; ADR, *P* = 0.6597) (Table 4). Details of AEs are presented in Additional file 2: Table S1.

## Discussion

Chronic gastritis with erosion (also referred to as “chronic erosive gastritis”) is a well-known lesion which is occasionally seen during routine endoscopy. Chronic erosive gastritis is a special variants of chronic gastritis according to the Sydney system. Erosive gastritis can be divided into two types: flat and bulged-shape.

The treatment effect of flat erosion and bulge-shaped one is different, especially in the case of bulged erosions. The bulged type is also known as endoscopic erosive gastritis, complete erosion, varioliform erosions, and, in Japan, “octopus sucker”; however, endoscopists often diagnose chronic gastritis with varioliform lesions. For this study, the type of erosion was randomly allocated as the influencing factor to ensure comparative objectivity so as to prevent the large deviation of the included patients among groups.

Despite still being unclear, the pathogenesis of erosive gastritis appears to be caused by disruptions of normal mucosal defense mechanism and reduced gastric mucosal blood flow caused by various endogenous and exogenous pathogenic factors, including gastric acid and pepsin. Gastric erosions may also be linked with *H. pylori* infection, drug stimulation, obesity, high body mass index, and bile reflux [9, 10, 31–33]. As has been reported, *H. pylori* infection, allergic respiratory diseases, high levels of work-related stress, irregular meals, spicy food consumption, pickled food consumption in older people, and excessive smoking in men all have been positively correlated with this condition’s incidence [34].

In this study, *H. pylori*-infected patients were specifically excluded, since the erosion related to this infection can often be resolved following the eradication of *H. pylori* by quadruple therapy [35, 36]. Our objective was to determine the efficacy of TCM in treating patients with erosion caused by other factors rather than *H. pylori* infection; however, this poses certain limitations in evaluating the overall effectiveness of TCM in erosive gastritis.

It is possible to see erosion in chronic superficial gastritis and chronic atrophic gastritis. The former has a short course of disease and can be treated easily, while the latter has a longer course and a poorer outcome associated with treatment.

A possible association between gastric varioliform lesions, namely the bulged type of chronic erosive gastritis, and gastric neoplasia has been established in some studies. Munoz Monteavaro et al. [37] described a case of “in situ” carcinomatous transformation in gastric varioliform lesions in 1960. In a retrospective case–control study conducted in China involving 1638 chronic gastritis patients, Zou et al. [34] reported that mucosal atrophy, intestinal metaplasia and low-grade dysplasia were significantly higher in the case group, indicating a potential cancer risk associated with gastric varioliform lesions. Another retrospective study conducted in Spain examined 42 patients with chronic gastritis with varioliform lesions and found that 86% had intestinal metaplasia and 31% had dysplasia or early neoplasia [38]. The authors concluded that gastric varioliform lesions are easily detected by endoscopy, which may assist endoscopists to detect an early development of neoplastic lesions in the gastric mucosa [38]. The bulged lesions can continue to grow and transform into sessile polyps that later present as a gastric carcinoma; therefore, the World Congress of Gastroenterology (WCOG) classified the disease as a precursor to gastric cancer. Compared with normal gastric mucosa, gastric varioliform lesions exhibited significantly higher levels of thioredoxin domain-containing protein 5 (TXNDC5) [39]. It was concluded that increased expression of TXNDC5 can result in increased proliferation and invasive activity, suggesting that it may serve as an oncogene and contribute to gastric carcinogenesis [39]. Hence, regular endoscopic follow-ups are an integral part of the treatment of chronic gastritis with varioliform lesions.

As indicated by “*Consensus of clinical experts on gastrointestinal mucosal protection (2021, Fuzhou)*” [40], mucosal protective agents are as effective as PPIs in treating chronic gastritis with erosion associated with various etiologies; however, mucosal protective agents are more effective than PPIs in relieving symptoms such as abdominal distension and belching. Therefore, it is justified to use an antacid as a positive control for chronic atrophic gastritis with erosion and aluminum plus magnesium suspension was chosen according to previous literature [27–29].

Mucosal protective compounds also include sucralfate, guaiazulene, alginates, and other antacids. Particularly, sucralfate, a compound consisting of aluminum hydroxide and sucrose sulfate, rapidly forms a strong and viscous mucosal barrier in the gastrointestinal tract, which is commonly used for healing mucosal erosions and relieving symptoms [41]. Despite its frequent use in clinical practice, aluminum plus magnesium suspension has been less well studied regarding its mechanisms of mucosal protection. It has been shown that sucralfate can reverse gastrointestinal damage caused by aspirin [42]. In a rat model, sucralfate had a greater anti-inflammatory response compared with omeprazole and suppressed the mucosal increase in endothelin-1 more effectively [43]. The use of sucralfate in combination with other agents is also used to treat gastric dysplasia [44]. Further studies are therefore awaited to examine the efficacy of Chinese herbal compound prescriptions together with *Panax notoginseng* powder in treating chronic gastritis with erosion using sucralfate as the control group.

There were two test groups that received Chinese herbal compound prescriptions plus *Sanqi* (*Panax notoginseng*) powder, or plus *Sanqi* (*Panax notoginseng*) powder and *Zhebeimu* (*Fritillaria thunbergii* Miq.) powder. According to the theory of TCM, *Sanqi* has a variety of medicinal properties, including the ability to dissipate blood stasis and arrest bleeding, to reduce swelling, and to relieve pain. The effects of *Zhebeimu* include clearing heat, resolving phlegm, relieving coughing, removing toxins, dispelling toxins, and dissolving carbuncles. Phlegm and blood stasis are the principal pathogenic factors associated with mucosal erosions. By dispersing blood stasis and resolving phlegm with herbal powder, this pathogenesis can be addressed. In this regard, herbal powder mimics the gastric mucosal protective agent (i.e., aluminum plus magnesium suspension) that has topical effects on the mucosa. Besides, the study design based on eight TCM prescriptions for the treatment of the corresponding TCM syndrome emphasizes the holistic concept and the principles of syndrome differentiation and treatment, which aimed to examine whether herbal prescriptions would alleviate symptoms and improve pathological scores for atrophy, intestinal metaplasia, and dysplasia.

Endoscopy performed at 4 weeks of treatment is planned to evaluate the improvement of erosions for this study. To increase compliance of patients and ensure the operability of the study, the scope of the study was narrowed down to patients with chronic atrophic gastritis with erosions.

Chronic gastritis with erosions belongs to the category of “epigastralgia”, “stomach heaviness”, and “gastric upset” in TCM literature. In TCM theory, a syndrome pattern plays an important role in evaluating pathogenesis. There is a certain regularity in this condition’s TCM syndrome distribution. Studies have shown that there are statistical differences among TCM syndromes in erosive gastritis, and the frequency of the syndrome types varies from high to low, including syndrome of spleen-stomach dampness-heat, syndrome of stagnant heat in liver and stomach, syndrome of stagnation of liver-qi, syndrome of blood stasis in stomach collaterals, syndrome of stomach-yin deficiency, and syndrome of spleen and stomach deficiency-cold; TCM syndrome is also related to *H. pylori* infection and gastric mucosal atrophy, and syndrome of spleen-stomach dampness-heat is more prevalent in *H. pylori* infection [45]. This study showed that the most common syndromes associated with erosive gastritis were syndrome of spleen-deficiency and qi-stagnation, syndrome of spleen-stomach dampness-heat, syndrome of stagnation of liver-qi, syndrome of stagnant heat in liver and stomach, and syndrome of spleen and stomach deficiency-cold. These results were consistent with previous research.

This study has shown that both aluminum plus magnesium suspension and Chinese herbal compound prescriptions together with *Panax notoginsen*g powder are capable of improving gastric mucosal erosion and reducing gastric mucosal pathological scores, and there were no statistically significant differences among the groups in primary endpoints, indicating that TCM therapy can achieve similar efficacy than antacids in terms of primary outcomes. The aluminum plus magnesium suspension is superior to the TCM therapy in improving acute inflammation of the gastric mucosa, is comparable to TCM in improving atrophy and intestinal metaplasia, and is inferior to TCM in improving dysplasia. In addition, the TCM therapy significantly outperforms the aluminum plus magnesium suspension in improving symptoms. Therefore, the overall clinical outcome of Chinese herbal compound prescriptions together with *Panax notoginseng* powder based on TCM syndrome differentiation in the treatment of erosive gastritis is superior to that of antacids.

As the first multi-center randomized controlled study on herbal medicine to assess its efficacy and safety in treating chronic atrophic gastritis with erosion, this study examines eight types of herbal compound prescriptions available to patients with chronic erosive gastritis based on eight types of TCM syndromes; however, the molecular mechanisms by which these prescriptions treat erosive gastritis have not been fully examined, which may require further investigation.

Due to the lack of precise localization markers in the gastric mucosa, it is quite easy to cause a deviation of the localization and miss the diagnosis of lesions in re-examining ordinary biopsy samples. The mucosa marking targeting biopsy (MTB) technique is performed with a specific calibration biopsy forceps and marked with the same staining point, which is convenient for re-examination and identification and therefore objective for assessing the results of chronic gastritis treatment [46]. In this study, the MTB technique was not used to measure the effect before and after treatment. However, the total score and the highest score were used to evaluate and analyze the gastric mucosa histological score. The results were consistent, which suggests the findings were objective.

Accordingly, the Chinese herbal compound prescriptions together with *Panax notoginseng* powder based on traditional Chinese medicine syndrome differentiation is a safe and effective treatment for chronic atrophic gastritis with erosion. It is more effective than antacids in improving clinical symptoms, and demonstrate a certain superiority in the treatment of gastric mucosal atrophy, intestinal metaplasia and dysplasia.

## Supplementary Information

**Additional file 1: Fig. S1.** The pathological efficacy rate of gastric mucosa in each group (total score). **Fig. S2.** The pathological efficacy rate of gastric mucosa in each group (highest score).

**Additional file 2: Table S1.** Details of adverse events.

## Data Availability

The datasets used in the present study are available from the corresponding author on reasonable request.

## Abbreviations

ADRs: Adverse drug reactions
AEs: Drug-related adverse events
ANCOVA: Analysis of covariance
ANOVA: Analysis of variance
CI: Confidence intervals
CMH: Cochran-Mantel–Haenszel
FAS: Full analysis set
*H. Pylori*: *Helicobacter pylori*
LSM: Least-square means
SD: Standard deviation
MTB: Mucosa marking targeting biopsy
PPIs: Proton pump inhibitors
PRO: Patient-reported outcome
TCM: Traditional Chinese medicine
UTI: Urinary tract infection
