# Supplementary material for: Chinese herbal compound prescriptions combined with Chinese medicine powder based on traditional Chinese medicine syndrome differentiation for treatment of chronic atrophic gastritis with erosion: a multi-center, randomized, positive-controlled clinical trial
Source: Chin Med. 2022 Dec 22;17:142. doi: 10.1186/s13020-022-00692-7 (PMC9773465; doi:10.1186/s13020-022-00692-7)
Supplement: Supplementary file 1 — Additional file 1: Fig. S1. The pathological efficacy rate of gastric mucosa in each group (total score). Fig. S2. The pathological efficacy rate of gastric mucosa in each group (highest score). [file 13020_2022_692_MOESM1_ESM.docx]

**
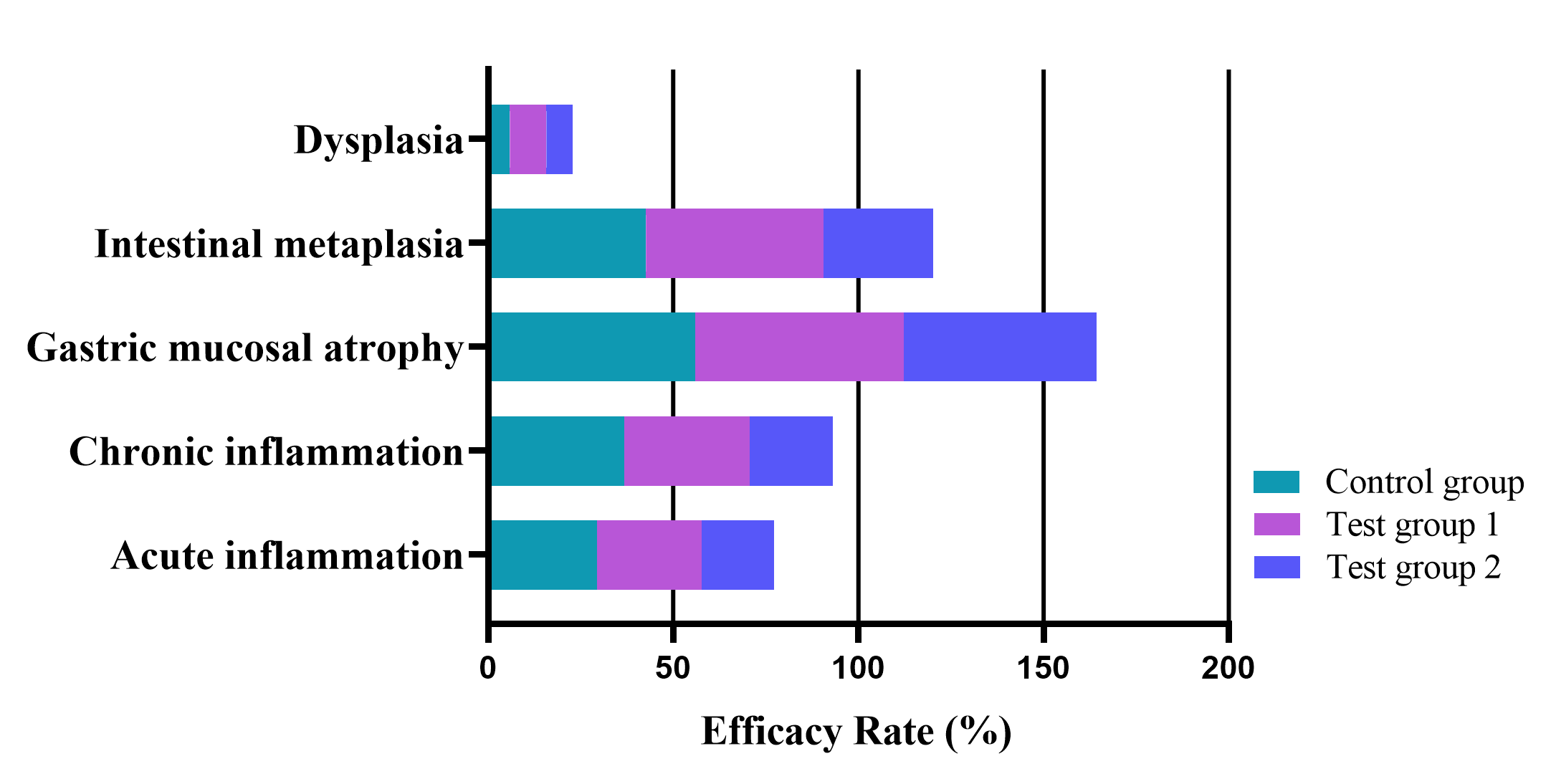
**

**Fig. S1** The pathological efficacy rate of gastric mucosa in each group (total score)


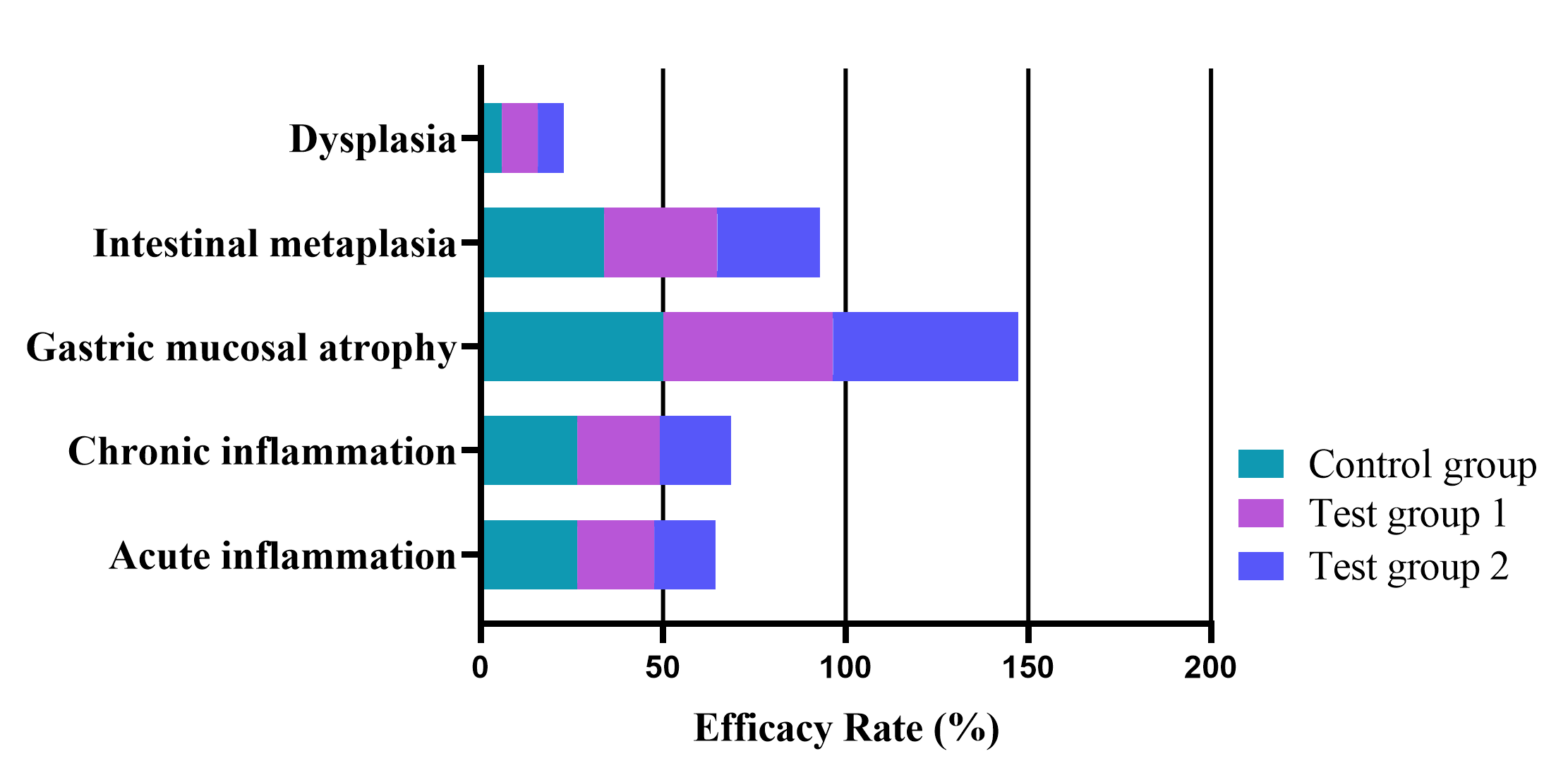


**Fig. S2** The pathological efficacy rate of gastric mucosa in each group (highest score)
