# Supplementary material for: Chinese herbal compound prescriptions combined with Chinese medicine powder based on traditional Chinese medicine syndrome differentiation for treatment of chronic atrophic gastritis with erosion: a multi-center, randomized, positive-controlled clinical trial
Source: Chin Med. 2022 Dec 22;17:142. doi: 10.1186/s13020-022-00692-7 (PMC9773465; doi:10.1186/s13020-022-00692-7)
Supplement: Supplementary file 2 — Additional file 2: Table S1. Details of adverse events. [file 13020_2022_692_MOESM2_ESM.docx]

**Table S1** Details of adverse events

| Subject number | Clinical center number | AE | AE start date | AE end date | Severity of AE | Serious AE | Action taken with study drug | AE outcome | Is AE treated with symptomatic treatment | Has the  patient discontinued the study because of an AE | Is AE related to the drug | Symptoms, signs, tests and the treatment of the ADRs or AEs |
| --- | --- | --- | --- | --- | --- | --- | --- | --- | --- | --- | --- | --- |
| 90 | 3 | UTI | 2017-02-21 | 2017-02-25 | Mild | No | Discontinuation | Recovered | No | No | Likely | There were no obvious symptoms, and the urine white blood cell level was high. After a period of drug withdrawal, the patient's urine routine was not abnormal, and no additional treatment was required. |
| 101 | 3 | UTI | 2017-05-05 | 2017-05-15 | Mild | No | Discontinuation | Recovered | No | No | Likely | This patient has no symptoms and the urine test shows a high level of white blood cells. After a period of drug withdrawal, the urine routine will return to normal and no special treatment is required. |
| 116 | 2 | Abnormal liver function | 2017-06-27 |  | Mild | No | Not applicable | Remission | Pharmacological treatment | No | Likely | Following treatment, transaminase increased and then decreased after drug withdrawal. |
| 117 | 3 | Fever | 2017-07-03 | 2017-07-06 | Mild | No | Discontinuation | Recovered | No | No | Suspicious | A blood test was performed following the patient's treatment, and a medical history was requested. The patient complained that he had symptoms of fever for the past two days, and other symptoms were not evident. The patient was instructed to drink more water and take proper rest. |
| 135 | 6 | Possible UTI | 2017-09-29 |  | Mild | No | Dose remained unchanged | Unknown | No | No | Unlikely | Following treatment, a routine examination of the patient's urine revealed that the urine white blood cells were slightly elevated, 15-20/HPF. Possible UTI was considered; however, the patient had no symptoms of urinary tract discomfort or fever and was advised to drink more water. |
| 200 | 4 | Urticaria | 2017-11-13 |  | Mild | No | Discontinuation | Remission | No | Yes | Suspicious | After taking the drug for three days, the patient reported urticaria recurrence, associated with itching, and the urticaria improved following discontinuation of the drug as recommended by the dermatologist. |
